# Accurate Prediction of Children's ADHD Severity Using Family Burden Information: A Neural Lasso Approach

**DOI:** 10.3389/fncom.2021.674028

**Published:** 2021-06-21

**Authors:** Juan C. Laria, David Delgado-Gómez, Inmaculada Peñuelas-Calvo, Enrique Baca-García, Rosa E. Lillo

**Affiliations:** ^1^Department of Statistics, University Carlos III of Madrid, Madrid, Spain; ^2^Santander Big Data Institute, Universidad Carlos III de Madrid, Madrid, Spain; ^3^Department of Psychiatry, Fundación Jiménez Díaz Hospital, Madrid, Spain; ^4^Department of Psychiatry, Nimes University Hospital, Nimes, France

**Keywords:** deep learning, lasso, feature selection, interpretability, ADHD

## Abstract

The deep lasso algorithm (dlasso) is introduced as a neural version of the statistical linear lasso algorithm that holds benefits from both methodologies: feature selection and automatic optimization of the parameters (including the regularization parameter). This last property makes dlasso particularly attractive for feature selection on small samples. In the two first conducted experiments, it was observed that dlasso is capable of obtaining better performance than its non-neuronal version (traditional lasso), in terms of predictive error and correct variable selection. Once that dlasso performance has been assessed, it is used to determine whether it is possible to predict the severity of symptoms in children with ADHD from four scales that measure family burden, family functioning, parental satisfaction, and parental mental health. Results show that dlasso is able to predict parents' assessment of the severity of their children's inattention from only seven items from the previous scales. These items are related to parents' satisfaction and degree of parental burden.

## 1. Introduction

Attention-deficit hyperactivity disorder (ADHD) is the most common chronic psychiatric disorder in childhood (Wender and Tomb, 2016). According to a recent systematic review, this neurodevelopmental disorder has an estimated prevalence in children and adolescents of 7.2% (Thomas et al., 2015). ADHD is characterized by inattention, excessive activity, and impulsive behavior. Children with ADHD have a higher risk of suffering from accidents, school failure or addiction problems (Harpin, 2005; Elkins et al., 2007). In addition, it has been observed that untreated children present low self-esteem and poor social functioning in the long term (Harpin et al., 2016). Fortunately, it has been observed that these negative consequences are reduced with an early and accurate diagnosis (Sonuga-Barke et al., 2011).

The diagnosis of ADHD is obtained through a clinical interview in which the clinician relies on the information provided by parents or teachers. However, several studies have shown that this information can be influenced by the characteristics of the informant. For example, it has been shown that female young teachers tend to provide more severe scores than older male teachers (Schultz and Evans, 2012). In another study, Chi and Hinshaw predicted the discrepancies between the reports provided by the mother and the teacher based on the mother's responses to the Beck Depression Inventory (Chi and Hinshaw, 2002). They observed that the responses provided by mothers with depression were negatively biased. This result has been validated in other studies (Harvey et al., 2013; Madsen et al., 2020). In addition, it has been observed that parental stress is another factor that explains the discrepancy between parents and teachers (Yeguez and Sibley, 2016; Chen et al., 2017).

This article, which expands the above studies, focuses on determining whether it is possible to predict the severity of a child's inattention and hyperactivity/impulsivity reported by his/her parents based on their distress and family burden. Furthermore, it seeks to identify the factors that influence parents' assessments. To achieve these objectives, the deep lasso (dlasso) algorithm is developed. This algorithm combines recent advances in the fields of machine learning and statistics: deep learning and the least absolute shrinkage and selection operator (lasso).

Without a doubt, deep learning has become one of the greatest advances in recent years (Goodfellow et al., 2016). Improvements in hardware, the availability of larger databases, and algorithmic advances have made possible to accurately build neural networks with more than one hidden layer. These deep neural networks have managed to solve problems that were previously unattainable in the fields of computer vision (Liu et al., 2020), natural language processing (Young et al., 2018) or speech recognition (Nassif et al., 2019).

However, in mental health, getting accurate results is not enough. Knowing the factors that characterize a given disease is often as important as precisely detecting those patients who suffer the condition. Identifying the relevant factors allows for improved treatments and prevention measures. One scientific field that has put a lot of effort in creating explainable models is the area of statistics. Among the techniques developed in this field, the lasso algorithm is undoubtedly one of the most widely used (Tibshirani, 1996). The lasso algorithm performs variable selection by including a regularization term in the loss function of the linear regression. The importance of this technique can be observed in the several extensions that have been developed to deal with variable selection. These techniques, which modify the loss function, include Elastic-Net (Zou and Hastie, 2003, 2005; Witten et al., 2014), Group Lasso (Zhou and Zhu, 2010; Zhao et al., 2014) or recently Sparse Group Lasso (Simon et al., 2013; Vincent and Hansen, 2014; Rao et al., 2016; Laria et al., 2019). However, in order to use these techniques, a database with a moderate/large size is needed since the regularization parameter is estimated through crossvalidation. Having a database of this size is not always possible in the mental health field.

The proposed dlasso algorithm, a neuronal network with two hidden layers and a regularization term, combines the advantages of lasso and the neural networks. On the one hand, like the lasso linear model, dlasso performs variable selection and provides the weights associated with each selected variable. This makes the neural network explainable. On the other hand, the weights of the neural network are trained through the backpropagation algorithm which, unlike traditional lasso, makes to automatically find the optimal value of the regularization parameter possible. Therefore, dlasso proposes to be a bridge that connects the prominent area of neural networks with that of modern statistics to obtain mutual benefits.

The rest of this article is organized as follows. Next section introduces the dlasso technique. Section 3 compares the performance of dlasso with respect to the traditional non-neural lasso. Additionally, some technical details of our implementation are highlighted in this section. After showing dlasso's performance, also in this section, it is used to predict children's ADHD severity based on their parent's burden and to identify the factors that influence parents' assessment. Finally, section 4 includes a discussion of the implication of our findings for future research.

## 2. Model formulation

Consider the usual linear lasso framework, where we have a data matrix ***X*** ∈ ℝ^*N*×*p*^ containing *N* observations of dimension *p*, a response vector ***y*** ∈ ℝ^*N*^, and the objective is to find ***β*** ∈ ℝ^*p*^ that minimizes, for some λ, the objective function

(1) $$L(\beta ,\lambda )=\sum_{i=1}^{N}{\left(y_{i}-\sum_{j=1}^{p}X_{ij}\beta_{j}\right)}^{2}+\lambda ||\beta |{|}_{1}.$$

where $\left\|\beta \right\|=\sum_{i=1}^{p}\left|\beta_{i}\right|$ is the *L*_1_ norm.

The general approach chooses the value of λ that minimizes the quadratic error term in L on a separate dataset, using some type of cross-validation over a grid (see, for example, Friedman et al., 2010; Friedman, 2012). In order to develop our methodology, the following proposition provides an alternative definition of the lasso problem.

**Proposition 1**. The lasso problem Equation (1) is equivalent to,

(2) $$\underset{w}{\min}\left\{\sum_{i=1}^{N}{\left(y_{i}-\frac{\lambda_{0}}{\lambda }\sum_{j=1}^{p}X_{ij}w_{j}\right)}^{2}+\lambda_{0}||w|{|}_{1}\right\},\text{ with }\lambda ,\lambda_{0}>0.$$

The proof of Proposition 1 is straightforward, taking ω = λ***β***/λ_0_. Although there is also λ_0_, the regularization hyper-parameter in Equation (2) is λ, because λ_0_ is a fixed constant. The hyper-parameter λ_0_ could be interpreted, to some extent, as an initial approximation of λ if we were solving the traditional lasso.

**Figure 1:**
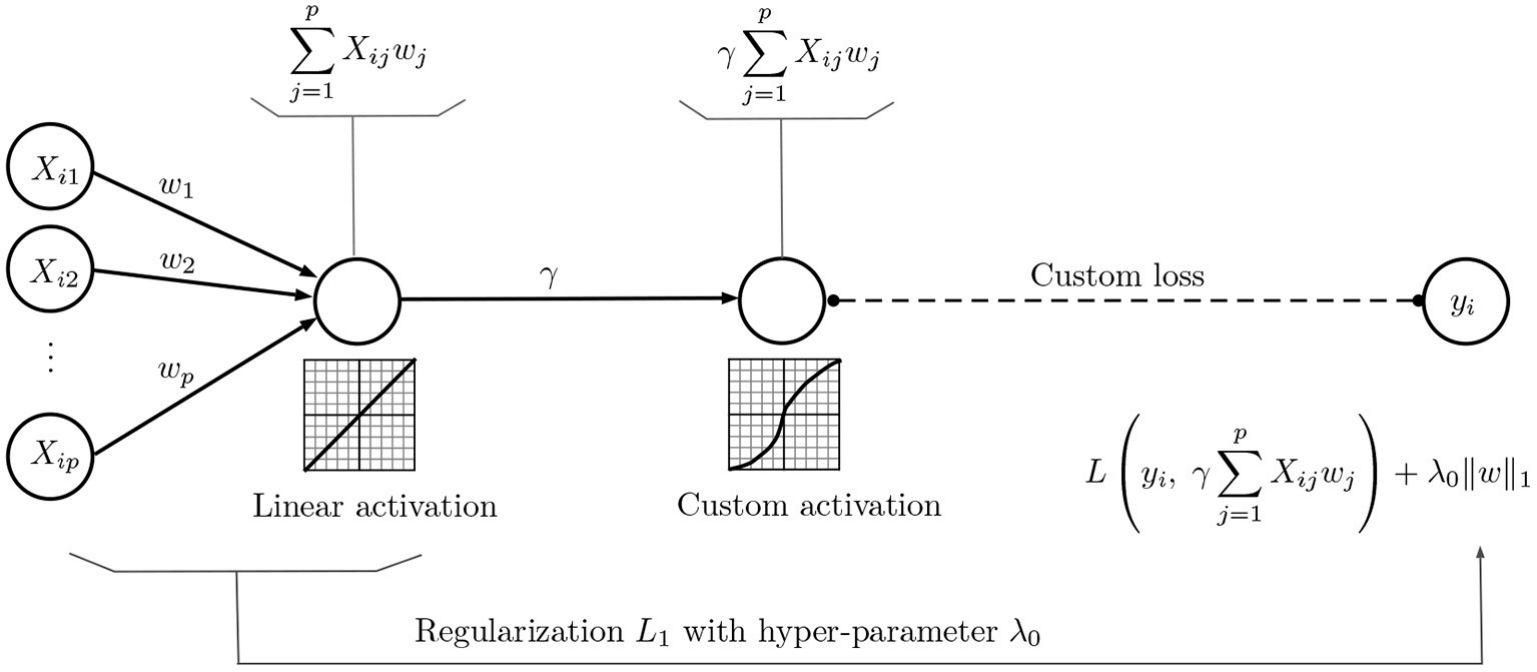
Neural network representation of the lasso problem Equation (2).

Based on Proposition 1, Problem Equation (2) can be formulated as the neural network of Figure 1, assuming that the weight γ = λ_0_/λ is constant. However, this neural representation is a more general approach than Equation (2), because the parameter γ is optimally selected as part of the training process, unlike previous methodologies that rely on some sort of cross validated set-up to select λ.

Regarding the weights' optimization, it is known that in the context of neural networks, *L*_1_ regularization does not completely zero out the weights. This is because the neural network optimizer does not take into account the non-differentiability of the regularization term at ω_*j*_ = 0. To carry out feature selection from the neuronal network perspective, a condition on the weights is imposed so that, after a given number of iterations, the weights that satisfy this condition are forced to be exactly 0. The mathematical derivation of the condition is presented from an optimization perspective, using subgradient conditions on problem Equation (2). General optimization problems where the objective function is the sum of a convex differentiable function (the squared error in this case) and a convex non-smooth part (the penalty) are discussed in Beck and Teboulle (2009a,b). In our case, it is simpler than that, since we are only interested in the condition that makes a particular ω_*j*_ = 0. Notice that automatic feature selection in our context means a solution to Equation (2) where ***w*** has many components that are exactly zero.

Assume that we have some estimation of ω, γ, which is the result of optimizing the weights in the neural network of Figure 1, after a number of epochs. Focusing on ω, and letting γ fixed, we have,

(3) $$\begin{array}{r} \underset{\omega }{\min}F\left(\omega \right):=||y-\gamma X\omega |{|}_{2}^{2}+\lambda_{0}||\omega |{|}_{1}. \end{array}$$

The optimality of any solution ω* of Equation (3) is characterized by the subgradient conditions. That is, for every *j* = 1, 2, …, *p*,

(4) $$0=\partial_{j}F(\omega^{*})=-2\gamma X_{j}^{T}(y-\gamma X\omega^{*})+\lambda_{0}v_{j},$$

where

$$v_{j}=\{\begin{array}{cc} sign(\omega_{j}^{*}) & \;\omega_{j}^{*}\neq 0\\ \in [-1,1] & \;\omega_{j}^{*}=0 \end{array}$$

In particular, $\omega_{j}^{*}=0$ if

$$0=-2\gamma X_{j}^{T}\left(y-\gamma \sum_{i=1,i\neq j}^{p}X_{i}\omega_{i}^{*}\right)+\lambda_{0}v_{j},\;|v_{j}|\leq 1,$$

which is equivalent to

(5) $$\left|2X_{j}^{⊤}\left(y-\gamma \sum_{i=1,i\neq j}^{p}X_{i}\omega_{i}^{*}\right)\right|\leq \left|\frac{\lambda_{0}}{\gamma }\right|.$$

Equation (5) provides a natural criterion to update the weights ω, trained after some number of epochs.

## 3. Experimental Results

In this section, the performance of dlasso is evaluated in two different scenarios that have been previously used in the literature.

### 3.1. Experiment 1

This first experiment is based on the one conducted in the original lasso article (Tibshirani, 1996). The data is simulated from the model ***y*** = ***Xβ*** + ϵ, where ϵ_*i*_ ~ *N*(0, 5) and

$$\beta =[3\;1.5\;0\;0\;2\;\underset{p-5}{\underset{︸}{0\ldots 0}}].$$

The data matrix ***X*** is simulated so that the correlation between its columns ***X***_*i*_ and ***X***_*j*_ is given by $\rho_{ij}=0.5^{\left|i-j\right|}$, for 1 ≤ *i* < *j* ≤ *p*. To illustrate different configurations, the number of variables *p* varies in {20, 100, 200}. In addition, in order to obtain significant results, the simulation for each configuration is repeated 100 times.

The training data set was composed of 50 observations, whereas 950 observations were used to test the performance of lasso and dlasso. Unlike dlasso, which automatically tunes the regularization parameter along with the optimization of the coefficients, the ***β*** estimation provided by the lasso method depends on a user-supplied value of λ. This hyper-parameter was optimally selected using random search on a grid of size 1, 000, compared across 5 bootstrap repetitions of the training data. To fit the lasso model, and select the hyper-parameter λ, we used the following R libraries: glmnet (Friedman et al., 2010) for the model engine, parsnip (Kuhn and Vaughan, 2020) for the tidymodel interface, and tune (Kuhn, 2020) for tuning λ using random search.

Regarding the fit of the dlasso's parameters, the algorithm was trained for 1,000 epochs. In order to avoid initialization dependence, the training process was repeated 20 times and the network with the minimum training loss (mean squared error) was selected as the final model.

Table 1 summarizes the simulation results for Experiment 1, displaying the root mean squared error (rmse), the ***β*** recall (proportion of true non-zero coefficients correctly identified) and the ***β*** precision (proportion of zero coefficients correctly identified), of both lasso and dlasso. The values reported in Table 1 are averaged over the 100 repetitions, with the corresponding standard deviations in parenthesis. We performed paired *t*-tests to evaluate the significance of the differences between both methods, denoting with (**) those for which the *p*-value was lower than 0.01 and (*) if the *p*-value was lower than 0.05. This table shows that the proposed dlasso method obtains a lower rmse than lasso in all the scenarios. Furthermore, this difference seems to be accentuated as the number of noise variables in the problem increases.

**Table 1:** Simulation results of the first experiment.

	**Method**	**rmse**	**recall (β)**	**precision (β)**
*p* = 20	dlasso	0.5(0.08)	1(0)	0.659(0.18)^**^
	lasso	0.5(0.08)	1(0)	0.486(0.19)
*p* = 100	dlasso	0.518(0.08)^**^	0.99(0.06)	0.348(0.13)
	lasso	0.531(0.09)	0.99(0.06)	0.362(0.22)
*p* = 200	dlasso	0.545(0.11)^**^	1(0)^*^b	0.244(0.08)
	lasso	0.561(0.11)	0.99(0.06)	0.313(0.19)^**^

*Differences at the 0.05 and 0.01 significance levels are denoted by*

* and ** *, respectively*.

### 3.2. Experiment 2

The second experimental set-up is based on the simulation studies carried out by Witten et al. (2014). The data is simulated according to the linear model ***y*** = ***Xβ*** + ϵ, with the number of features *p*, and ϵ_*i*_ i.i.d. from a *N*(0, 10) distribution (1 ≤ *i* ≤ *n*). The data matrix ***X*** is simulated from a multivariate *N*(**0**, **Σ**) distribution, where **Σ** ∈ *R*^*p*×*p*^ is block diagonal, given by

$$\Sigma ={\left[\begin{array}{ccc} \Sigma_{\rho } & \;0 & \;0\\ 0 & \;\Sigma_{\rho } & \;0\\ 0 & \;0 & \;0 \end{array}\right]}_{p\times p},$$

with $\Sigma_{\rho }\in R^{20\times 20}$ such that

$$\Sigma_{\rho }(i,j)=\{\begin{array}{cc} 1 & \;i=j\\ \rho & \;i\neq j \end{array},$$

with ρ denoting the correlation inside groups. The true coefficient vector ***β*** ∈ *R*^*p*^ is also random, given by,

$$\beta =[\beta_{1}\;\beta_{2}\;\ldots \;\beta_{10}\;\underset{10}{\underset{︸}{0\;\ldots \;0}}\;\beta_{21}\;\beta_{22}\;\ldots \;\beta_{30}\;\underset{p-30}{\underset{︸}{0\;\ldots \;0}}],$$

where

$$\beta_{j}\sim \{\begin{array}{ll} U[0.9,1.1], & 1\leq j\leq 10\\ U[-1.1,-0.9], & 21\leq j\leq 30 \end{array}.$$

To explore different scenarios, the parameter ρ ∈ {0, 0.5, 0.8}, whereas *p* ∈ {40, 100, 400}, resulting in a total of 9 possible configurations. Similarly to the previous experiment, the data is composed of 50 and 950 observation for training and test, respectively, and the simulation for each configuration is repeated 100 times. The estimation of ***β*** and the selection of λ is carried out as described in the previous experiment.

Table 2 illustrates the results that were obtained by each methods in the different configurations. As before, the values reported in Table 2 are averaged over the 100 repetitions, with the corresponding standard deviations in parenthesis, and significant differences at level 0.01 are denoted with (**). It is observed that dlasso obtains better results as the number of variables increases, which suggests that dlasso might be a more suitable approach in the high-dimensional setting.

**Table 2:** Simulation results of the second experiment.

	**Method**	**rmse**	**recall (β)**	**precision (β)**
	ρ = 0.1			
*p* = 40	dlasso	0.73(0.1)	0.684(0.1)	0.748(0.1)^**^
	lasso	0.701(0.11)^**^	0.804(0.14)^**^	0.679(0.11)
*p* = 100	dlasso	0.797(0.1)^**^	0.612(0.1)	0.526(0.09)^**^
	lasso	0.838(0.15)	0.624(0.23)	0.469(0.15)
*p* = 400	dlasso	0.875(0.13)^**^	0.511(0.12)^**^	0.275(0.06)
	lasso	0.939(0.16)	0.332(0.24)	0.399(0.2)^**^
	ρ = 0.5			
*p* = 40	dlasso	0.454(0.07)	0.722(0.09)	0.689(0.08)^**^
	lasso	0.401(0.05)^**^	0.784(0.08)^**^	0.658(0.07)
*p* = 100	dlasso	0.477(0.08)	0.71(0.11)	0.655(0.09)^**^
	lasso	0.476(0.14)	0.75(0.12)^**^	0.463(0.12)
*p* = 400	dlasso	0.494(0.08)^**^	0.73(0.12)	0.539(0.1)^**^
	lasso	0.588(0.27)	0.74(0.17)	0.33(0.17)
	ρ = 0.8			
*p* = 40	dlasso	0.344(0.05)	0.772(0.1)^**^	0.597(0.07)
	lasso	0.302(0.04)^**^	0.637(0.09)	0.634(0.08)^**^
*p* = 100	dlasso	0.356(0.05)	0.747(0.1)^**^	0.584(0.08)^**^
	lasso	0.347(0.1)	0.627(0.13)	0.451(0.13)
*p* = 400	dlasso	0.368(0.06)^**^	0.782(0.09)^**^	0.558(0.06)^**^
	lasso	0.425(0.22)	0.652(0.18)	0.354(0.18)

*Differences at the 0.05 and 0.01 significance levels are denoted by*

* and ** *, respectively*.

## 4. Computational Issues

The dlasso algorithm has been implemented in R, using the keras library (Allaire and Chollet, 2019), and TensorFlow as backend (Abadi et al., 2016). Concerning the optimizer, we have chosen ADAM (Kingma and Ba, 2014), but our theoretical model formulation is not limited to a particular optimizer, and in this regard, different configurations may be tested.

**Figure 2:**
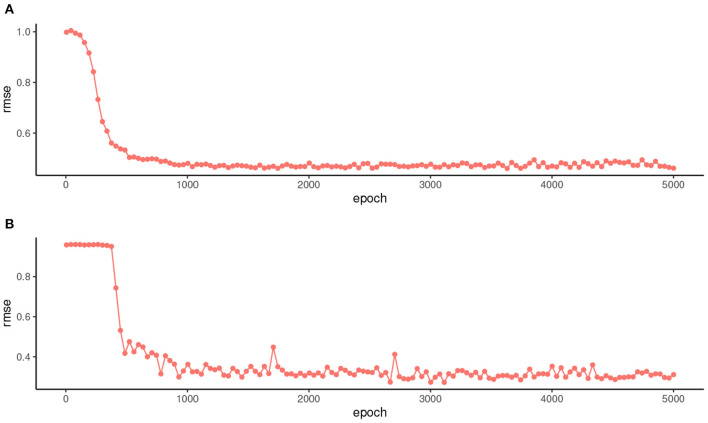
Evolution of the rmse in the test data over 5,000 iterations for one of the simulations of the first experiment **(A)** and for one of the second experiment **(B)**.

An important computational issue is how to set the number of iterations to train the network, since a very large number can degrade its performance on future data. Figure 2 shows how the rmse evolves in the test data over the different iterations for one of the simulations of the first experiment (Figure 2A) and for one of the second experiment (Figure 2B). These two plots are representative of the different simulations. In both cases, no performance drop is observed in the test data as the number of iterations increases. This shows that the proposed method is quite robust to variations in the number of epochs and that, for example, 1,000 iterations is an acceptable value in both experiments.

### 4.1. Experiment 3

Once that the performance of the proposed dlasso technique has successfully been evaluated, this third experiment examines whether it is able to predict the assessments that parents make about the severity of their children's ADHD symptoms based on their distress and family burden.

To this end, the parents of 73 children diagnosed with ADHD by the medical professionals at the Fundacioń Jimeńez Díaz Hospital of Madrid participated in this study. The parents (54 mothers and 19 fathers) were required to sign an informed consent after the study was explained in detail to them. The mean age of their children was 12.4 years. The consent form and the study protocol were reviewed and approved by the Institutional Review Board of Fundacioń Jimeńez Díaz Hospital (reference: EO 77/2013_FJD_HIE_HRJC).

The participants assessed the severity of their children's symptoms through the Strengths and Weaknesses of ADHD-symptoms and Normal-behavior (SWAN) scale (Swanson et al., 2012). The SWAN scale is composed of 18 items, based on the DSM-5 criteria, for ADHD diagnosis which measure positive attention and impulse regulation behaviors in the normal population. The first nine items measure inattention while the remaining nine assess impulsivity/hyperactivity. The sum of the first nine items is used as an indicator of the severity of inattention, while the sum of the last nine items is used as an indicator of the severity of impulsivity/hyperactivity. The family burden and distress were assessed with the following scales:

- **The Zarit Burden Interview**. This 22-item self-report inventory examines the burden associated with functional/behavioral impairments and the home care situation (Zarit and Zarit, 1987; Schreiner et al., 2006). The responses ranged from never to always. It has been pointed out that this instrument has an excellent internal consistency (Bédard et al., 2001).
- **The General Health Questionnaire-12 (GHQ-12)**. This questionnaire, consisting on 12 items, measures general mental health (Anjara et al., 2020). The responses ranged from much worse than usual to better than usual. Sanchez-Lopez and Dresch showed that, in a Spanish sample of 1001 participants, the GHQ-12 exhibited an adequate reliability and external validity (Sánchez-López and Dresch, 2008). They also indicated that the GHQ-12 is an efficient technique to detect non-psychotic psychiatric problems.
- **The family Adaptability, Partnership, Growth, Affection, and Resolve (Apgar) scale**. This five-item scale is used to assess how family members perceive the level of functioning of the family unit (Smilkstein, 1978). Responses ranged from hardly ever to almost always.
- **Visual Analoge Scale (Vas) on life satisfaction**. This is an *ad hoc* questionnaire designed by the Department of Translational Psychiatry of the Fundación Jiménez Díaz Hospital and which is part of the electronic questionnaires administered by a digital tool. Parents were asked to rate their own level of satisfaction in different life areas: themselves, family, friends, work and leisure activities. Parents scored these aspects on a scale from 0 to 10, where a higher number means more satisfaction.

Once the data were collected, a repeated validation analysis was conducted to test whether the dlasso algorithm was able to predict the severity of parent-reported child inattention, via the SWAN scale, based on the responses that they provided to the 44 previous predictors. The number of repetitions was set to 100. For each repetition, and similarly to the previous experiments, the training set contained 50 randomly selected observations. The remaining 23 observations were used to evaluate the performance of dlasso. The number of training epochs was 1,000. The performance measures were the rmse and the correlation between the total scores obtained with the SWAN inattention subscale and the values predicted by dlasso. The average correlation obtained by dlasso was 0.34 (std: 0.15) and the rmse was 1.99 (std: 2.87). These results indicate that dlasso was able to obtain good estimates of parents' assessment of their children's inattention. In addition, it was also observed that the correlation and rmse that would have been obtained if a multiple linear regression was applied were –0.01 (std: 0.23) and 4.14 (std: 14.12), respectively. These numbers reflect the importance of conducting feature election.

The previous repeated validation study was replicated, but using parent-reported child hyperactivity as the dependent variable. However, unlike the previous results, dlasso was not able to accurately estimate hyperactivity with these predictors. The average correlation obtained by dlasso was 0.03 (std: 0.19) and the rmse was 2.35 (std: 3.69). Similar results would have been obtained if all predictors were included in the multiple linear regression. Concisely, the average correlation would have been –0.05 (std: 0.20) and the rmse 4.34 (std: 17.6). These results show that, for our data, it is possible to estimate the degree of children's inattention through parental reported distress and family burden, but not the severity of children's hyperactivity.

**Figure 3:**
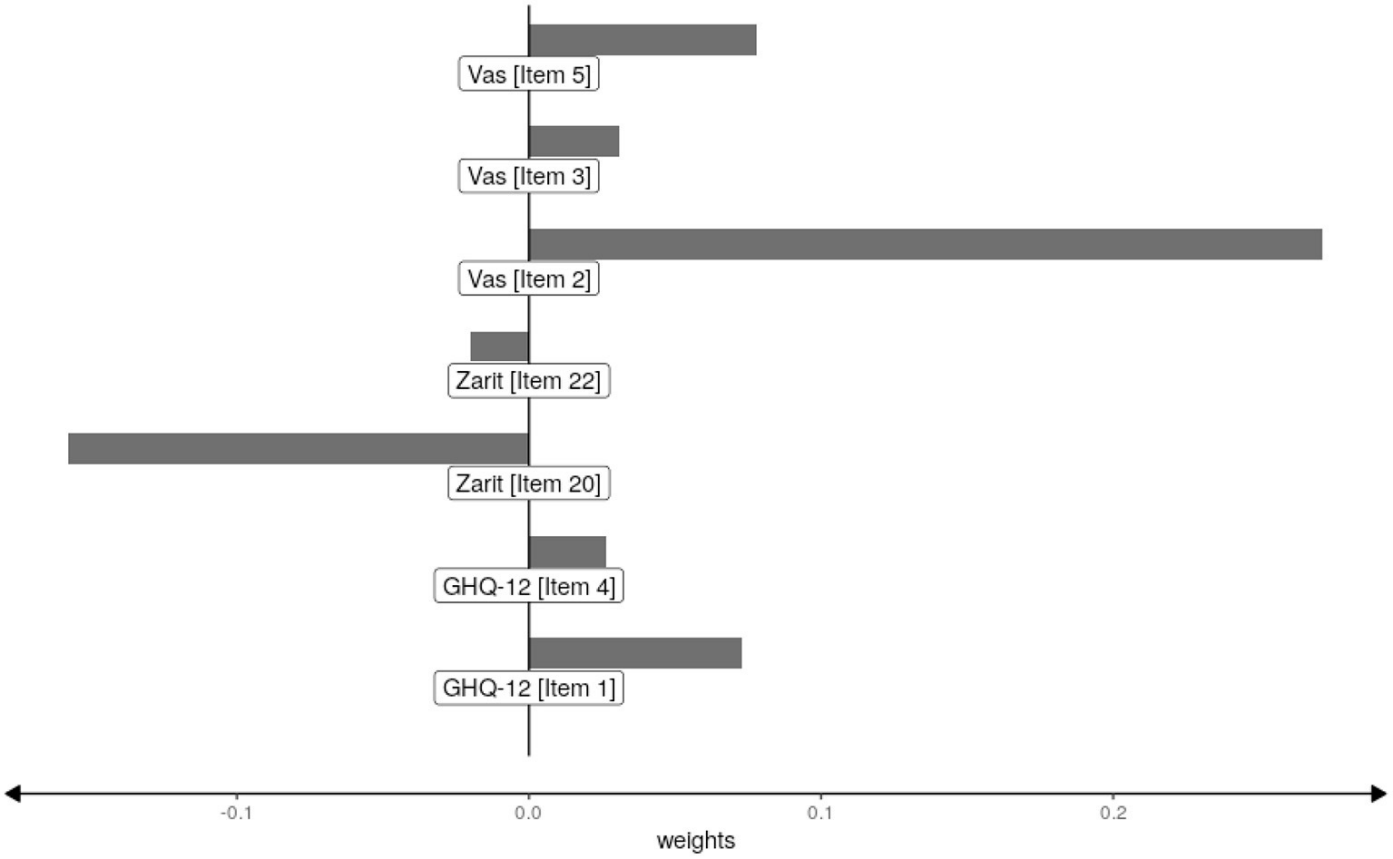
Weights of the selected items.

After evaluating the performance of the proposed technique, a third analysis was carried out to determine which items dlasso used to estimate inattention. To do this, the dlasso was run on the whole sample and with all the predictor variables. The different predictors were standardized so that the selected items could be compared based on the absolute value of their weights. The variables selected were:

- GHQ-12, Item 1: Have you recently been able to concentrate on what you're doing?
- GHQ-12, Item 4: Have you recently felt capable of making decisions about things?
- Zarit, Item 20: Do you feel you should be doing more for your relative?
- Zarit, Item 22: How burdened do you feel in caring for your relative?
- Vas, Item 2: Satisfaction with Family
- Vas, Item 3: Satisfaction with Friends
- Vas, Item 5: Satisfaction with Leisure Activities

and the adjusted *R*^2^ was 0.314. It is observed that only 7 of the 44 items were used to make the predictions. It is also noted that no items of the Apgar scale were selected. The values of the weights of the selected items are shown in Figure 3.

This figure shows that the most influential items are the second item of the Vas scale and item 20 of the Zarit scale. Both items are related to family satisfaction. It is also observed that three of the five items of the Vas scale, about the person's satisfaction with friends, pleasure activities and family, are selected.

## 5. Conclusions

In this article, the dlasso method has been proposed, implementing the well-known lasso feature selection technique using neural networks. The performance of the proposed dlasso has been assessed in two experiments previously referenced in the literature. In most of the conducted simulations, the proposed dlasso has attained a lower rmse, and significant higher precision and recall in the variable selection than the traditional lasso. Moreover, the simulation studies reveal that the gap between dlasso and its traditional counterpart widens as the number of variables increases.

In a third experiment, dlasso was used to predict the severity of symptoms in children with ADHD from the responses provided by their parents to four questionnaires aimed at measuring family burden, family functioning, parental satisfaction, and parental mental health. It was observed that dlasso was able to predict the severity of inattention using only seven items out of the 44 available. Interestingly, three of these seven items were obtained from the life satisfaction scale. Specifically, it was observed that higher parental satisfaction in essential domains such as family, friends and leisure activities are good predictors of inattentive symptomatology in children. The remaining four items are related to anxious symptomatology. Another noteworthy issue is that the algorithm did not select any of the items of the Apgar scale, which implies that family functioning is not taken into account in predicting inattention. This result complements the findings reported in the literature that show parental stress as one of the factors of disagreement among informants. Specifically, van der Oord et al. showed that parental stress explained 12% of the variance in the disagreement of parent and teacher ratings of inattention (van der Oord et al., 2006). Subsequently, Yeguez and Sibley showed that parental stress, maternal education, and maternal ADHD predicted high maternal grades relative to teacher-reported (Yeguez and Sibley, 2016). van der Veen-Mulders et al. also showed that differences in ratings between fathers and mothers were due to parental stress (van der Veen-Mulders et al., 2017).

The results obtained, together with those reported in the literature, raise the question of whether the stress reported by parents is mostly caused by their children's symptoms or whether it is caused by external factors. Several studies have pointed to the first hypothesis. In this case, parental stress could be used as an excellent predictor of the severity of their children's ADHD symptoms. However, on the other hand, stress could also be caused mostly by external factors. Therefore, these results point to the need to establish mechanisms that identify the source of parental stress so that the relevance of the evaluation carried out by them can be assessed.

Regarding hyperactivity/impulsivity, dlasso was not able to obtain accurate estimates. Children diagnosed with ADHD inattentive subtype are usually diagnosed later than those with hyperactive/impulsive and/or combined subtypes (Milich et al., 2001). This may lead to significant family overload, psychological distress and poorer family functioning.

These results build a bridge between statistical and artificial intelligence approaches that allows tackling mental health conditions in which large samples are difficult to obtain.

## Data Availability Statement

The datasets presented in this article are not readily available because the data are protected by the hospital as they refer to minors. Requests to access the datasets should be directed to ebacgar2@yahoo.es.

## Ethics Statement

The studies involving human participants were reviewed and approved by Institutional Review Board of Fundacioń Jimeńez Díaz as meta Hospital. Written informed consent to participate in this study was provided by the participants' legal guardian/next of kin.

## Author Contributions

JL and DD-G: conceptualization and formal analysis. JL, DD-G, and RL: methodology. JL: software. JL, DD-G, RL, and IP-C: writing–original draft preparation. RL and EB-G: supervision. DD-G: funding acquisition. All authors contributed to the article and approved the submitted version.

## Conflict of Interest

The authors declare that the research was conducted in the absence of any commercial or financial relationships that could be construed as a potential conflict of interest.
